# Association between Sleep Quality and Duration and Periodontal Disease among University Students: A Cross-Sectional Study

**DOI:** 10.3390/ijerph17093034

**Published:** 2020-04-27

**Authors:** Md Monirul Islam, Daisuke Ekuni, Naoki Toyama, Ayano Taniguchi-Tabata, Kota Kataoka, Yoko Uchida-Fukuhara, Daiki Fukuhara, Hikari Saho, Nanami Sawada, Yukiho Nakashima, Yoshiaki Iwasaki, Manabu Morita

**Affiliations:** 1 Department of Preventive Dentistry, Okayama University Graduate School of Medicine, Dentistry and Pharmaceutical Sciences, Okayama 700-8558, Japan; p3a99o50@s.okayama-u.ac.jp (M.M.I.); pu171qxi@s.okayama-u.ac.jp (N.T.); de18017@s.okayama-u.ac.jp (K.K.); de422026@s.okayama-u.ac.jp (H.S.); de422027@s.okayama-u.ac.jp (N.S.); mmorita@md.okayama-u.ac.jp (M.M.); 2 Department of Preventive Dentistry, Okayama University Hospital, Okayama 700-8558, Japan; de19026@s.okayama-u.ac.jp (A.T.-T.); de20006@s.okayama-u.ac.jp (Y.U.-F.); de20041@s.okayama-u.ac.jp (D.F.); nakashima109@okayama-u.ac.jp (Y.N.); 3 Department of Oral Morphology, Okayama University Graduate School of Medicine, Dentistry and Pharmaceutical Sciences, Okayama, 700-8558, Japan; 4 Health Service Center, Okayama University, Okayama 700-8530, Japan; yiwasaki@okayama-u.ac.jp

**Keywords:** sleep quality, sleep duration, periodontal disease, university students

## Abstract

The purpose of this cross-sectional study was to investigate the association between sleep quality and duration, and periodontal disease among a group of young Japanese university students. First-year students (*n* = 1934) at Okayama University who voluntarily underwent oral health examinations were included in the analysis. Sleep quality and duration were assessed by the Japanese version of the Pittsburgh Sleep Quality Index. Dentists examined Oral Hygiene Index-Simplified (OHI-S), probing pocket depth (PPD), and percentage of sites with bleeding on probing (BOP). Periodontal disease was defined as presence of PPD ≥ 4 mm and BOP ≥ 30%. Overall, 283 (14.6%) students had periodontal disease. Poor sleep quality was observed among 372 (19.2%) students. Mean (± standard deviation) sleep duration was 7.1 ± 1.1 (hours/night). In the logistic regression analysis, periodontal disease was significantly associated with OHI-S (odds ratio [OR]: 2.30, 95% confident interval [CI]: 1.83–2.90; *p* < 0.001), but not sleep quality (OR: 1.09, 95% CI: 0.79–1.53; *p* = 0.577) or sleep duration (OR: 0.98, CI: 0.87–1.10; *p* = 0.717). In conclusion, sleep quality and duration were not associated with periodontal disease among this group of young Japanese university students.

## 1. Introduction

Sleep has been considered an important element for health. In Japan, the percentage of young adults (20–29 years) with inadequate rest was higher than that of all Japanese (24.3% vs. 21.7%, respectively), and a national survey showed that this ratio is increasing [1]. Poor sleep is related to decreased immunity and increased inflammatory status. These conditions influence the occurrence and progression of several diseases including diabetes mellitus, metabolic syndrome, hypertension, stroke, and coronary artery disease [2,3,4,5].

Periodontal disease is a widespread multifactorial chronic inflammatory disease, comprising inflammatory conditions that affect surrounding tooth structures (e.g., gingiva, bone, and periodontal ligament), that subsequently leads to tooth loss [6]. There are still unknown risk factors for periodontal disease and further studies are required. Furthermore, it is very important to control risk factors for periodontal disease at an early stage, especially for young adults, because more than 30% of young adults in Japan have periodontal disease and the prevalence increases with aging [7]. An animal study suggested that deep sleep deprivation increases alveolar bone loss and gingival inflammation [8]. Recent cross-sectional studies suggested that poor sleep quality and/or duration are associated with the prevalence of periodontitis [9,10,11,12,13,14]. However, a nationally representative study of participants aged ≥ 30 years reported that there is no significant association between periodontal disease and poor sleep [15]. Therefore, the association between sleep quality and duration and periodontal disease remains unclear. Furthermore, little is known about such association in Japanese.

In other countries, sleep quality among university students was previously assessed and a high prevalence of impaired sleep quality (59.4%–60.4%) was observed among them [16,17]. Thus, young adults, especially university students, are one of the important targets of research. We hypothesized that sleep quality and duration in university students are associated with periodontal disease. Therefore, this cross-sectional study aimed to determine the association between sleep quality and duration, and periodontal disease among young Japanese university students.

## 2. Materials and Methods

### 2.1. Study Participants

In this cross-sectional study, we collected data from first-year students who underwent general health examinations at the Health Service Center of Okayama University in April 2018 based on the research target. The inclusion criteria were students who volunteered to receive an optional oral examination and completed a questionnaire. The exclusion criteria comprised incomplete data, age >19 years, current smoker, daily alcohol drinker, and having systemic diseases.

### 2.2. Ethical Procedures and Informed Consent

Informed consent was verbally obtained from all participants before the study was started. The present study was approved by the Ethics Committee of Okayama University Graduate School of Medicine, Dentistry, and Pharmaceutical Sciences (No. 1060). Our study conformed with Strengthening the Reporting of Observational Studies in Epidemiology (STROBE) guideline.

### 2.3. Self-Questionnaire

A questionnaire was mailed to all participants before the health examination. Items included age, sex, oral health behaviour (e.g., daily frequency of tooth brushing, use of dental floss, and visits to a dental clinic [18]), smoking habit, alcohol drinking habit, having systemic diseases, and exercise habit.

### 2.4. Assessment of Sleep Quality

Sleep quality was assessed using the Japanese version of the Pittsburgh Sleep Quality Index (PSQI-J) [19]. The PSQI-J is a valid, reliable, and standardized self-reported questionnaire to measure sleep quality over the last month. Previously, it was used to assess the sleep quality of Japanese young adults [20]. The PSQI-J comprises seven component scores (range of subscale scores: 0–3) as follows: (i) sleep quality, (ii) sleep latency, (iii) sleep duration, (iv) habitual sleep efficiency, (v) sleep disturbances, (vi) use of sleeping medication, and (vii) daytime dysfunction. Subscale scores of all components were summed to yield a global score (range: 0–21). Higher PSQI scores indicate poorer sleep quality. In this study, poor sleep quality was defined as PSQI-J score > 5 [21]. Sleep duration (hours/night) was calculated using responses from the questionnaire.

### 2.5. Assessment of Stress

Psychological stress was measured using the 10-item Perceived Stress Scale (PSS-10) [21]. In this study, we used the Japanese version of PSS-10, which has been used for the study of stress among Japanese [22]. Moreover, PSS-10 has also been used to assess psychological stress among young university students in particular [23]. The PSS-10 consists of a 10-item questionnaire; six items are positively phrased and four items are negatively phrased. Response options are rated on a five-point Likert scale ranging from 0 (never) to 4 (very often). Responses to all items are then summed to calculate the total score, which ranges from 0 to 40. Higher scores indicate higher perceived stress. In the present study, scores were grouped into the following categories of perceived stress: 27–40, high stress; 14–26, moderate stress; and 0–13, not exposed to stress.

### 2.6. Oral Examination

Individuals underwent a complete oral examination by seven trained dentists (D.E., K.K., A.T-T., Y.U-F., D.F., N.T., and H.S.). The tooth number was enumerated, and then periodontal condition was assessed using the Community Periodontal Index (CPI) [18]. For periodontal examination, 10 teeth were selected (molars in each posterior sextant, and the upper right and lower left central incisors). A CPI probe (YDM, Tokyo, Japan) was used to measure each tooth at six sites (mesiobuccal, mid-buccal, distobuccal, distolingual, mid-lingual, and mesiolingual) for measurement of probing pocket depth (PPD) and bleeding on probing (BOP). PPD (≥ 1mm) is the depth of gingival sulcus and reflects the status of periodontal disease. Higher PPD indicates worsened periodontal disease. BOP is an earlier and more sensitive indicator of inflammation than visual signs of inflammation (e.g., redness and swelling) [24]. Therefore, we calculated the percentage of teeth exhibiting BOP among the 10 examined teeth (0–100%) [18]. Higher percentage of BOP indicates higher inflammatory activity. The level of dental plaque and calculus was measured to assess the simplified oral hygiene index (OHI-S) [25]. Higher OHI-S score indicates poorer oral hygiene. Seven experienced dentists were trained to determine PPD in the 10 teeth evaluated by CPI and measurements were repeated after a 2-week interval in two volunteers. Good intra- and inter-examiner agreement for the oral examination was acquired (kappa value > 0.80.)

### 2.7. Statistical Analysis

Power calculations were conducted using G*Power (ver. 3.1.9.2, Universität Kiel, Kiel, Germany), considering mean PSQI score as the primary outcome from a previous study [9]. The minimum sample size required for our study was 45 to yield a power of 95% and α of 0.05. During the study, we had a sufficient number of students. However, to obtain effective results, we considered more participants for the analysis. We confirmed the normality of data by histogram, *Q-Q* plots, and the Shapiro–Wilk test. In this study, we defined periodontal disease as PPD ≥ 4 mm and BOP ≥ 30% (active periodontal pocket) [26]. Continuous variables are expressed as means and standard deviations (SDs), while categorical variables are shown as percentiles in descriptive analysis. Continuous variables were analysed by the *t*-test or Mann–Whitney *U* test, whereas categorical variables were analysed by the chi-squared test. Following the logistic regression model, we calculated the odds ratio (OR) and 95% confidence interval (CI). In all analyses, *p*-value < 0.05 was considered statistically significant. Data analysis was performed using SPSS (v. 25.0; SPSS Inc., Chicago, IL, USA).

## 3. Results

**Figure 1:**
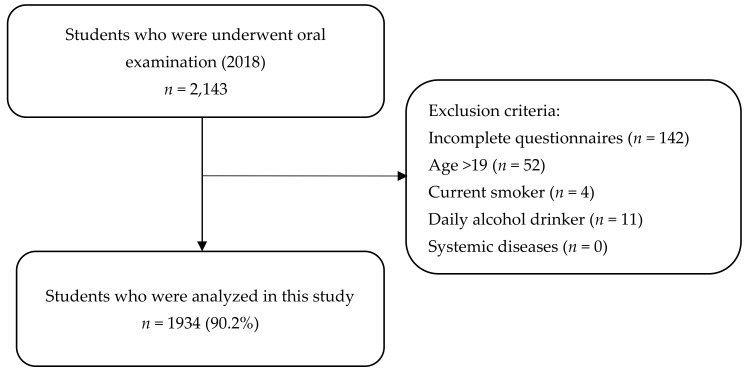
Recruitment flowchart.

The recruitment flowchart is shown in Figure 1. Ultimately, 1934 students (1058 men; 876 women) met all inclusion and exclusion criteria, and were included in the analysis. Demographic characteristics of the study participants are presented in Table 1. Overall, 283 (14.6%) participants had periodontal disease. Poor sleep quality was identified among 372 (19.2%) participants. The mean sleep duration was 7.1 hours/night.

Table 2 shows a comparison between the periodontal disease and non-periodontal disease groups. The number of male participants was significantly higher in the periodontal disease group than the non-periodontal disease group (*p* < 0.05). Furthermore, the periodontal disease group had significantly higher mean OHI-S and tooth number than the non-periodontal disease group (*p* < 0.05). However, there were no significant differences observed in sleep quality (PSQI) and duration between the two disease groups.

A logistic regression analysis showed that periodontal disease was not associated with sleep quality and sleep duration (*p* > 0.05 for both) (Table 3).

## 4. Discussion

To the best of our knowledge, our study is the first to examine the association between sleep quality and duration, and periodontal disease among young Japanese university students. Overall, our results show that sleep quality and duration were not significantly associated with periodontal disease in this group of young Japanese university students.

Several cross-sectional studies have reported an association between sleep quality and duration, and periodontal disease among different age groups (mean age in each group; ≥ 30 years) [9,10,11,12,13,14]. However, our results coincided with those of only one previous report [15]. The discrepancies between the results of our study and those of previous studies may be explained by differences in targeted age groups (young adults aged 18–19 years vs. adults aged ≥ 30 years). There might be the association between sleep quality and duration, and periodontal disease among older population in Japan. Moreover, in this study, we excluded a very small number of smokers and alcohol drinkers because there is evidence of influence of smoking and drinking on sleep quality and duration [27,28]. By contrast, some previous studies treated these behaviours as covariates [9,10,11,14].

We should pay attention to the definition of periodontitis. In this study, we defined periodontal disease as PPD ≥ 4 mm and BOP ≥ 30% (active periodontal pocket) [26]. We also evaluated the association between periodontal disease defined by only PPD ≥ 4 mm (CPI score = 3 or 4) and sleep quality and duration, as defined by a previous study [9]. However, no significant association was observed (data not shown). The prevalence of CPI = 4 (severe periodontitis) among university students was lower in the present study compared to that previously reported [9] (0.5% vs. 4.4%, respectively). Therefore, our findings must be confirmed in a greater number of Japanese young adults.

The PSQI global score (mean ± SD: 4.01 ± 1.95) of our study participants (18–19 years) was comparable with that reported by other studies. Previous studies from other countries have reported higher PSQI scores (mean, 5.6–6.6) [9,29,30,31]. The PSQI score in this study was lower than those of the previous studies. Thus, caution is needed when considering the generalizability of our findings.

In this study, we treated sleep duration and periodontal disease as continuous variables to investigate their association. However, some previous studies used a cut-off value of 7.0 hours/night for sleep duration, which indicated a short duration sleeper [9,11,14,15]. We also evaluated the association between periodontal disease and sleep duration using the cut-off value of 7.0 hours/night. However, there was no significant association (data not shown).

The findings of our study showed that the OHI-S was significantly higher among the periodontal disease group than the non-periodontal disease group. Moreover, in logistic regression analysis, OHI-S was identified as a risk factor for the periodontal disease group. This result is in line with a previous study among Japanese young adults [32]. Hence, university students should be encouraged to maintain good oral hygiene to minimize or control periodontal disease.

Psychological stress was not associated with periodontal disease among our study participants. Almost all students (95.2%) reported moderate stress in this study. A previous study showed a direct association between psychological stress and periodontal disease [33]. Although psychological stress was assessed by different instruments in different populations, the discrepancy may be associated with a ceiling effect.

The strengths of our study include the large number of participants, oral examination by calibrated dentists, and use of validated instruments. Furthermore, participants had no systemic diseases, smoking habit, or drinking habit. Characteristics of the targeted students were fairly homogeneous, which may have advantages for investigating the direct association between sleep quality and duration and periodontal disease.

There are some limitations associated with the present study. Firstly, this was a cross-sectional study. Therefore, an exploration of causation was not possible. Secondly, we did not examine other confounding factors such as body mass index [32], night-time computer and television use [17], tea consumption [34], skipping breakfast habit [35], and socio-demographic factors [36]. Thirdly, we selected only 10 teeth for periodontal disease screening. Therefore, the periodontal disease status could be different in our evaluation as compared with full mouth examination. Fourthly, the prevalence of periodontal disease (14.6%) was relatively low compared to the control (85.4%) and the participants were not equally recruited to give greater statistical power. However, the prevalence in this study was within the range of previous studies [7,26] and the number of participants was enough to perform the multivariate data analysis. Thus, the limitation may be small in this study. Finally, participants were enrolled from a single institution, which may limit the generalizability of our findings.

## 5. Conclusions

In conclusion, sleep quality and duration were not significantly associated with periodontal disease among this group of young Japanese university students.

## Figures and Tables

**Table 1:** Characteristics of participants.

Variables (*n* = 1934)	*n* (%)Mean ± SD
Gender	
Male	1058 (54.7)
Female	876 (45.3)
Age (y)	18.2 ± 0.4
Regular dental check-up (Yes)	572 (29.6)
Daily flossing (Yes)	499 (25.8)
Daily tooth brushing (≥2 times)	179 (9.2)
OHI-S	0.5 ± 0.5
Number of teeth present	28.3 ± 1.4
Exercise (Yes)	1462 (75.6)
Sleeping duration (hour/night)	7.1 ± 1.1
PSQI Global	4.0 ± 1.9
Sleep quality	
Poor (PSQI ≥ 5)	372 (19.2)
Good (PSQI < 5)	1562 (80.8)
PSS-10 (Total)	17.2 ± 2.5
Psychological stress	
No stress	87 (4.5)
Moderate stress	1843 (95.2)
High stress	4 (0.2)
Periodontal disease (Yes)	283 (14.6)

OHI-S, Simplified Oral Hygiene Index; PSQI, Pittsburgh Sleep Quality Index; PSS-10, The 10-item Perceived Stress Scale.

**Table 2:** Comparison between periodontal disease and non-periodontal disease groups.

Variables (*n* = 1934)	Periodontal Disease (*n* = 283)	Non-Periodontal Disease (*n* = 1651)	*p*-Value
Gender (Male)	185 (65.4) ^1^	873 (52.8)	<0.001 ^3^
Age (y)	18.2 ± 0.4 ^2^	18.2 ± 0.4	0.125 ^4^
Regular dental check-up (Yes)	82 (28.9)	490 (29.7)	0.811 ^3^
Flossing daily (Yes)	61 (21.6)	438 (26.5)	0.077 ^3^
Daily tooth brushing (≥2 times)	30 (10.6)	149 (9.0)	0.398 ^3^
OHI-S	0.7 ± 0.7	0.5 ± 0.5	<0.001 ^4^
Number of teeth present	28.7 ± 1.41	28.3 ± 1.35	<0.001 ^4^
Exercise (Yes)	222 (78.4)	1240 (75.1)	0.227 ^3^
Sleeping duration (hour/night)	7.0 ± 1.1	7.1 ± 1.2	0.283 ^4^
Sleep quality			
Poor (PSQI >5)	59 (20.8)	313 (18.9)	0.456 ^3^
PSQI Global	4.1 ± 2.0	3.9 ± 1.9	0.201 ^5^
PSQI domain score			
Sleep quality	1.0 ± 0.6	1.0 ± 0.6	0.868 ^4^
Sleep latency	0.8 ± 0.8	0.8 ± 0.8	0.145 ^4^
Sleep duration	0.9 ± 0.8	0.8 ± 0.8	0.260 ^4^
Habitual sleep efficiency	0.2 ± 0.5	0.1 ± 0.4	0.054 ^4^
Sleep disturbance	0.5 ± 0.5	0.5 ± 0.5	0.213 ^4^
Medication	0.02 ± 0.2	0.04 ± 0.3	0.133 ^4^
Daytime dysfunction	0.7 ± 0.7	0.7 ± 0.7	0.685 ^4^
Total PSS-10	17.1 ± 2.6	17.2 ± 2.5	0.900 ^4^
Psychological stress			
No stress	18 (6.3)	69 (4.2)	0.219 ^3^
Moderate stress	264 (93.3)	1579 (95.6)	
High stress	1 (0.4)	3 (0.2)	

OHI-S, Simplified Oral Hygiene Index; PSQI, Pittsburgh Sleep Quality Index; PSS-10, The 10-item Perceived Stress Scale; ^1^ *n* (%); ^2^ Mean ± SD; ^3^ Chi-square; ^4^ Mann–Whitney *U* test, ^5^ Unpaired *t*-test.

**Table 3:** Conditional odds ratios (ORs) and 95% confidence intervals (CIs) for periodontal disease.

Independent Variables		OR (95% CI) ^1^	*p*-Value
Gender	Female	Ref	0.015
	Male	1.42 (1.10–1.87)	
Age		1.28 (0.91–1.78)	0.156
Regular dental check-up	Yes	Ref	0.402
	No	0.88 (0.66–1.18)	
Flossing daily	Yes	Ref	0.153
	No	1.26 (0.92–1.73)	
Daily tooth brushing frequency	≥2 times	Ref	0.904
	<2 times	1.03 (0.67–1.59)	
OHI-S		2.30 (1.83–2.90)	<0.001
Exercise	Yes	Ref	0.522
	No	0.90 (0.65–1.24)	
Sleep quality	Good	Ref	0.577
	Poor	1.09 (0.79–1.53)	
Sleeping duration (hour/night)		0.98 (0.87–1.10)	0.717
Psychological stress	No stress	Ref	
	Moderate stress	0.63 (0.37–1.09)	0.103
	High stress	1.20 (0.11–12.77)	0.879

OHI-S, Simplified Oral Hygiene Index; ^1^ Adjusted for gender, age, regular dental check-up, flossing daily, daily tooth brushing frequency, OHI-S, exercise, sleep quality and psychological stress.
